# CAR T-Cell Production Using Nonviral Approaches

**DOI:** 10.1155/2021/6644685

**Published:** 2021-03-27

**Authors:** Viktor Lukjanov, Irena Koutná, Pavel Šimara

**Affiliations:** ^1^Masaryk University Brno, Faculty of Medicine, Department of Histology and Embryology, Kamenice 5, Brno 62500, Czech Republic; ^2^St. Anne's University Hospital Brno, International Clinical Research Center, Pekarska 53, Brno 656 91, Czech Republic

## Abstract

Chimeric antigen receptor T-cells (CAR T-cells) represent a novel and promising approach in cancer immunotherapy. According to the World Health Organization (WHO), the number of oncological patients is steadily growing in developed countries despite immense progress in oncological treatments, and the prognosis of individual patients is still relatively poor. Exceptional results have been recorded for CAR T-cell therapy in patients suffering from B-cell malignancies. This success opens up the possibility of using the same approach for other types of cancers. To date, the most common method for CAR T-cell generation is the use of viral vectors. However, dealing with virus-derived vectors brings possible obstacles in the CAR T-cell manufacturing process owing to strict regulations and high cost demands. Alternative approaches may facilitate further development and the transfer of the method to clinical practice. The most promising substitutes for virus-derived vectors are transposon-derived vectors, most commonly sleeping beauty, which offer great coding capability and a safe integration profile while maintaining a relatively low production cost. This review is aimed at summarizing the state of the art of nonviral approaches in CAR T-cell generation, with a unique perspective on the conditions in clinical applications and current Good Manufacturing Practice. If CAR T-cell therapy is to be routinely used in medical practice, the manufacturing cost and complexity need to be as low as possible, and transposon-based vectors seem to meet these criteria better than viral-based vectors.

## 1. Evolution of T-Cells with Chimeric Antigen Receptors

### 1.1. CAR T-Cells: Brief Definition

Technology based on chimeric antigen receptors (CARs) enables us to generate T-cells targeting virtually any existing protein structure on any cell. T-cells are genetically engineered to express a CAR that recognizes antigens on cancer cells. CAR T-cells then identify cancer cells and eliminate them from the organism. The specificity of this tumor target antigen is critical since its expression in healthy tissues might lead to severe side effects and even death.

### 1.2. History and Breakthroughs

In 1989, Zelig Eshar [1] and his team published the proof of concept of CAR T-cell therapy: first, T-cells were generated that expressed chimeric receptors that were activated after contact with target cells bearing the corresponding antibody. Remarkably, these CAR T-cells acted in a nonmajor histocompatibility complex- (MHC-) restricted manner. Arguably, the most important CAR T-cell clinical trial was performed in 2011 by Carl June's research group [2, 3]. They used a CAR against B-cell antigen CD19 to treat chronic lymphoid leukemia (CLL) in 3 patients. The CAR T-cells expanded 1000-fold after retransfusion, eliminated lymphoma cells, and induced complete and sustained remission. Although CAR T-cell-based technologies are still in development, the first therapeutic products have found their way into clinical practice. Kymriah (Novartis) and Yescarta (Gilead-KitePharma) were both approved by the Food and Drug Administration (FDA) for the treatment of B-cell-related malignancies. Both Yescarta and Kymriah target CD19. Among hematological oncogenic diseases, the treatment of B-cell malignancies has been shown to be the most successful. Although the therapy itself may cause B-cell aplasia because healthy B-cells are also affected, this state is clinically manageable.

Currently, there are 5 recognized generations of CAR T-cells with every new generation enhancing properties of the CAR construct by modification of its domains. Second-generation CAR T-cells include various costimulation domains (CD28 [4] or 4-1BB [5]) that enhance the efficiency of constructed T-cells [6]. The third generation utilized cooperation between multiple costimulation domains (e.g., CD3*ζ* + CD28+4-1BB). Activation of the PI3K/AKT signaling pathway was likely responsible for the enhanced properties of third-generation CAR T-cells [7, 8]. The fourth generation of CAR T-cells or TRUCKs (T-cells redirected for universal cytokine-mediated killing) expanded the cytotoxic properties by their ability to express transgenic cytokines (e.g., IL-12 [9]). Expression of transgenic IL-12 is linked to the specific environment (proximity of targeted cancer cells) and therefore could be especially helpful in the treatment of solid tumors [10]. The fifth generation of CAR T-cells added other cytokine-expressing domains (IL-15 [11]; IL-18 [12]), and the ability to target multiple antigens—targeting both HER2 + IL13R*α*2—was successfully tested in animal models of glioblastoma to prevent tumor antigen escape [13].

### 1.3. Viral vs. Nonviral Methods of CAR T-Cell Preparation

One of the major aspects of CAR T-cell preparation is the selection of a suitable vector that will carry the CAR construct into the cells. The two most commonly used options for CAR T-cell generation are viral-based vectors (usually retro- or lentiviruses) or nonviral vectors, which are dominantly transposon-based for CAR T-cell construction.

#### 1.3.1. Viral-Based Vectors

Currently, the vast majority of CAR T-cell production relies on the transfer of genetic information into T-cells by viral vectors. Retroviral genes (gag, pol, env) in combination with inducible promoters enhance transduction rates and produce relatively large numbers of CAR+ T-cells (reviewed in [14]). Vector-based murine leukemia virus (MLV) is the most commonly used gamma retroviral vector (reviewed in [15]) and was successfully used in T-cell immunotherapy for severe combined immunodeficiency- (SCID-) X1 disease as a novel approach [16]. Although the immunodeficiency disease was successfully treated, T-cell-related leukemia emerged in some cases [16, 17]. Vectors derived from another retroviral family, *Lentivirinae*, have shown better integration properties than their gamma retroviral counterparts. Lentiviral vectors are able to target nondividing cells with relative ease [18], whereas in gamma retroviral vectors, the transduction rates into nondividing cells are significantly lower [19]. The improved biological safety of lentiviral vectors is due to different integration inclinations. Gamma retroviral vectors prefer to integrate into gene promoters, which may be the cause of the oncogenic properties previously described (reviewed in [20]).

Viral vectors may be highly efficient in CAR T-cell production, but several key features discourage their use in the clinic in favor of nonviral approaches. First, the possibility of oncogenic and mutagenic potential calls for a more stable vector to be ultimately used in the preparation of clinical-grade CARs. Second, the use of viruses in current Good Manufacturing Practice (cGMP) laboratories is burdened with a set of strict regulations, and nonviral methods of gene transfer may be more feasible for clinical-grade manufacturing. Third, lentiviral/retroviral transduction is limited by the size of transported DNA [21]. Finally, some of the other vector systems (e.g., those that utilize electroporation, lipofection, ultrasound, or magnetofection for transduction) significantly reduce the overall price of CAR T-cell preparation. In general, the manufacturing cost of viral vectors tends to be higher than that of their transposon-based counterparts because the manufacturing process of such vectors is considerably more demanding (reviewed in [22]).

#### 1.3.2. Nonviral Vectors

The most common alternatives to viral vectors are transposons. A variety of transposon-based systems have been reported for CAR T-cell production, and these systems provide safe and reliable DNA transfer into T-cells. The sleeping beauty (SB) transposon system is currently being used as a substitute for viral-based vectors in the preparation of, for example, CD19+ CAR T-cells [23], with reported antitumor activity both in vitro and in vivo [24]. The main strength of this approach lies in the overall better integration profile of the transduced genetic material. This improved integration is achieved due to the lower promoter activity of the integrated transposon [25]. SB systems also trigger far fewer epigenomic changes in the proximity of an integration site. A relatively low manufacturing cost is also an important factor that further improves the position of SB systems [26]. The main obstacle for SB transposon usage is its significantly lower transgenic material integration rate. With the further development of SB systems, the problem of lower integration was significantly reduced. SB11 is a prime example of this effort and demonstrates 100-fold higher transposition rates than the native SB transposon [27]. With other modifications to the SB transposon system, the SB100X system increased the transposition rates up to 100 times in comparison to the SB11 system [28].

The integration profile of the SB transposon is close to random. The SB system targets TA sites for its integration [29]. Compared to the viral vectors ([30]), the SB transposons repeatedly demonstrated no integration bias towards coding sequences [31, 32]. Although the SB integration profile could be considered biologically safe, the other transposon vectors demonstrated properties more similar to viral-based vectors [32].

Transposon Tol2 is another example of a successful transposon system that is suitable for the creation of CAR T-cells [33]. In comparison to the naïve SB transposon, Tol2 offers greater coding capability (100-200 kb) and sufficient transposition activity [34]. Whereas SB transposons preferred T-A base sites for their integration, Tol2 seemed to have random integration preferences [33].

The greatest competition for SB transposons is most likely the PiggyBac (PB) transposon system. Originally, the PB element was first described and derived from cells of the moth *Trichoplusia ni* [35]. Similar to previously mentioned transposons, the PB transposon uses a simple cut-and-paste mechanism to integrate itself into human cells. The integration sites are nonrandom because the PB transposon usually prefers TTAA sequences [36]. Mapping of the PB integration profile revealed similarities with gammaretroviral and lentiviral vectors [37] with insertion bias into expressed genes comparable to MLV retrovirus [38]. Compared to other (Tol2 and SB11) transposon systems, the PB transposon system demonstrated superior transposition activity in mammalian cells [39]. Higher transposition activity in various mammalian cells positions the PB transposon at the top of potential nonviral vectors for human cell transgenesis and the manufacture of CAR T-cells. Another essential ability of the vector is the size of the cargo capacity. Cancer cells have multiple mechanisms to avoid an immune response, and optimal CAR constructs need to overcome the majority of these mechanisms for therapy to be successful. The PB transposon system was proven to be able to transfer multiple genes into T-cells, hence making them more potent for cancer therapy [40]. Nakazawa's [40] study also proved the capability of the PB system to carry and transfer safety switches in the form of a suicide gene into human T-cells. The ability to turn off CAR T-cells in the patient's body in the case of severe cytokine release syndrome (CRS) is perhaps the most crucial safety feature of the technology [41]. A number of hyperactive mutant variants of the PB transposon were successfully isolated [42]. The 7PB variant was able to outperform both naïve PB transposons and SB100X transposons in terms of transposition activity [43]. Another example of a modified PB system is the “mouse codon-optimized PB transposase gene”—mPB [44]. PB systems were successfully used to generate CD19 CARs for hematological malignancies [45] as well as CARs against selected solid tumor antigens, such as CD73 [46], MSLN [47], EGFRvIII [48], and PSMA [49]. The comparison between viral vectors and the two most common transposon vectors is shown in Table 1.

Transposon-derived plasmid vectors are reliant on a system for their delivery into the cell. Recently, the most commonly used method has been cell electroporation, with the main benefits being the relative simplicity of the procedure and the overall lower cost of the method. The electroporation of plasmid DNA is currently being surpassed by the electroporation of messenger RNA (mRNA). The main obstacle for plasmid DNA to enter the nucleus is the nuclear envelope. Therefore, dividing cells demonstrate much higher transduction rates than their nondividing counterparts [50]. With mRNA transfer, the need to overcome the nuclear envelope became redundant, although other problems emerged (mainly the decreased stability of the mRNA molecule compared to that of plasmid DNA). The lack of mRNA stability was significantly reduced by chemical modifications of the RNA nucleosides (e.g., the incorporation of pseudouridine [51]). Successful modifications of T-cells in mouse models by RNA electroporation were reported more than 15 years ago [52], and significant progress has been made in this regard since then [53, 54]. The preclinical testing of mRNA-mediated CARs showed the suitability of the method in the treatment of solid tumors [55, 56]. Aside from electroporation, the usage of lipid nanoparticles as a form of mRNA transport was recently demonstrated in the preparation of CAR T-cells [57]. Unlike electroporation, this method is significantly less toxic to the transduced cells.

Although both approaches are suitable for cGMP-quality CAR T-cell production, electroporation is being used far more frequently.

## 2. CARs in Practice: Clinical Applications

Clinical trials regarding CAR T-cell-based therapy are rapidly evolving, and an increasing number of clinical trial approval requests are submitted each year. By the end of 2016, 124 ongoing clinical trials of CAR T-cell therapies for hematological malignancies, and 57 clinical trials for solid tumors were registered worldwide [62]. The majority of these trials were performed in the US or in China. Less than 10% of these trials were performed in Europe. Since then, the situation has significantly evolved. There are more than 600 clinical trials in various stages of CAR T-cell therapies, according to ClinicalTrials.gov. The majority of clinical trials still focus on hematological malignancies (267 of these trials involve CARs that target CD19). The majority of ongoing clinical studies adopted viral-based vectors for CAR T-cell manufacture, although the portion of studies that adopted transposon vectors is steadily growing. Although concerns for possible vector-induced oncogenic activation have not yet been observed in clinical applications, theoretical risks are still presented [63]. LV systems used in the clinic are dominantly derived from HIV-1 virus. Several approaches have been implemented to reduce the biohazard properties of LV vectors [64]. A popular example of such modification usable in clinical practice is a four-plasmid system that is able to effectively split the HIV-1 genome, making viral gene expression dependent on different separated transcription units and genes responsible for packaging only expressible in producer cells (HEK293T cell lines are frequently used) [65]. Integration of multiple plasmids carrying parts of the LV vector ultimately increases the logistical complexity of larger-scale CAR T-cell manufacture; therefore, more optimized LV systems are still sought after [66]. The main clinical application for viral-constructed CARs remains in the treatment of blood-related malignancies [67–69].

### 2.1. Transposon Clinical Trials

Analogous to viral-based CARs, transposon-mediated CAR T-cell clinical applications also dominantly focus on blood cancers [23]. CD19-specific CAR T-cells transduced via SB vector already exhibited promising results during phase-I clinical trials [31, 70, 71]. A successful approach in the preparation of cGMP-grade CAR T-cells (for phase I/II clinical trials) is to utilize electroporation of SB system DNA plasmids and cocultivation of T-cells with inactivated aAPCs (artificial antigen-presenting cells). Although transposon-mediated transduction is less effective, a sufficient number (10^10^) of CAR T-cells (95% purity) was repeatedly prepared during 28-day culture [72]. Analyzing the SB integration profile of used CARs showed no apparent hot spots or integration bias in transplanted cells. Recently, preliminary data from phase I/II clinical trials have suggested the biological safety of donor-derived CD19+ CAR T-cells, providing an opportunity for other allogenic applications of transposon-based CAR T-cells [71]. However, the optimal dosage of administered CAR T-cells is crucial for securing the safety of the therapy, as higher doses of CAR T-cells lead to stage I and stage II CRS [71]. Another factor that complicates the severity of CRS is the severity of the disease itself. Using CAR T-cell therapy (aimed at blood cancers) in a short time period after the patient underwent hematopoietic stem cell transplantation could be beneficial for lowering the risk of CRS and ultimately eliminating residual disease from the patient [31]. Direct comparison of SB-mediated CAR T-cells in autologous and allogenic settings was also documented in the mentioned study [31]. Both approaches resulted in CAR T-cells of comparable purity, but autologous CAR T-cells were detectable for longer time periods in patients. Overall survival rates and 30-month progression-free rates were higher within the patients of the autologous trial, but both approaches resulted in significant improvements over standardly treated patients [73].

Although not as widespread as SB-CARs, PB-based CAR T-cells were also successfully constructed against CD19 antigen [74], with a phase I clinical trial being underway (ClinicalTrials.gov Identifier: NCT04289220).

## 3. CAR T-Cell Manufacturing

All of the previously mentioned methods of CAR T-cell preparation have their advantages, but the ultimate success of a certain approach is dependent on its reproducibility in strictly regulated cGMP conditions. These conditions determine the complex set of rules for the current manufacture of cellular therapies. Every aspect of CAR T-cell preparation needs to be well documented and in accordance with cGMP guidelines, starting with specifications for the cleanroom facility, where the manufacture is going to occur, to the emphasis on aseptic laboratory techniques, the content of culture media and other chemicals directly involved in the cultivation of the product and the processing of the final product (cryopreservation, quality control testing, etc.) (reviewed in [75]).

### 3.1. CAR T-Cell Cultivation

The cultivation process alone is considered to be a crucial part of the development of CAR T-cell-based therapeutics. To avoid contamination during cultivation, closed or semiclosed cultivation systems and bioreactors are considered to be superior to simple cultivation in a culture flask. G-Rex (Wilson Wolf Manufacturing) represents an example of a widespread semiclosed cell production system that utilizes cultivation in flasks with gas-permeable membranes, which enables better gas exchange and considerably enhances cell proliferation. Although the system offers upgrades from the usage of common cultivation flasks, it is not fully closed, and the potential risk of contamination could be higher than that of fully closed systems. Fully closed systems (e.g., CliniMACS Prodigy or Quantum Cell Expansion System) are able to perform the entire procedure (from cell transduction to expansion) within a single tubing set. The CliniMACS Prodigy tubing sets focus mainly on the preparation of CD19 CARs by the transduction of lentiviral vectors and subsequent activation by anti-CD3/CD28 antibodies [76]. When compared to the products of other established and conventional culture techniques, the final CAR T-cell product exhibited similar properties [77]. The Quantum Cell Expansion System is prevalently used for the manufacture of adherent cells such as mesenchymal stem cells (MSCs) [78], but the expansion of CAR T-cells was also proven possible [79].

Although fully closed systems minimize the likelihood of contamination, the limitations of viral-based vectors and their overall high cost considerably lessen their potential use in clinical practice. Current estimates of CAR T-cell therapy costs for a single patient range from $150 000 to 300 000 [63, 80]. Manufacturing cGMP quality CARs by plasmids/transposons is usually restricted to cultivation in open or semiopen culture systems because the electroporation (or lipofection) process is not automated yet in closed culture systems. CAR T-cells, transduced via electroporation, can be successfully prepared in cGMP conditions [81], although the aseptic technique demands on personnel are significantly higher.

To improve the posttransduction expansion and efficiency of T-cells, various cytokines are added to the culture media. The most commonly used cytokine for T-cell expansion promotion is probably IL-2 [82]. Although an increasing number of studies have suggested that a higher concentration of IL-2 could drive the CD8+ fraction of T-cells into terminal differentiation, this higher IL-2 concentration would not promote the formation of memory T-cells [83, 84]. Different interleukin combinations are therefore sought after to improve the properties of T-cell cultivation. To prevent T-cells from undergoing terminal differentiation, the combination of IL-7, IL-15, and IL-21 is frequently used [85]. IL-21 is similar to IL-2 in promoting CD8+ cytotoxic potential, although in an opposite manner. The addition of IL-21 inhibits CD8+ from terminal differentiation and could be regarded as an IL-2 antagonist [86]. IL-7 and IL-15 promote the formation of memory phenotypes within cultivated T-cells [87]. Concurrently, T-cell propagation in the presence of a combination of IL-7 and IL-15 led to more potent antitumor CARs than T-cell propagation in the presence of IL-2 [88].

In contrast to CAR T-cells targeted against cells of hematological malignancies, the viability and potency of CAR T-cells targeted against cells of solid tumors suffer in the proximity of the aggressive tumor microenvironment. To overcome this suppression, CARs can be modified with the so-called inverted cytokine receptor (ICR). Within the ICR, the interleukin-4 (IL-4) receptor is fused with the IL-7 receptor. This fused receptor is able to convert regulatory IL-4 signaling into IL-7 signaling and ultimately enhance the properties of CARs within the tumor microenvironment [89]. IL-4-supplemented culture media could increase the tumor-killing abilities of CAR T-cells [90].

Current trends in cGMP tend to avoid any animal-derived components in the cultivation process. The emphasis on xeno-free cultivation raises a concern regarding the culture media composition. Commonly used animal-derived components, such as fetal bovine serum (FBS), are preferably substituted by xeno-free alternatives. It was recently reported that substituting FBS with human platelet lysate had a positive effect on CAR T-cell conditions [91].

### 3.2. T-Cell Characterization

In addition to the transduction methods and specifics of different CAR structures, the characterization of modified T-cells should be addressed. The vast majority of studies simply utilize the CD3+ fraction of peripheral blood mononuclear cells (PBMCs) isolated from peripheral blood or leukapheresis. The composition of CD4+/CD8+ cells as well as the phenotypes of given cells is variable in most cases and studies, which could make it impossible to reproduce the results in a different environment. This obstacle could be solved with relative ease using advanced enrichment and cell-sorting technologies [92].

The CD4+/CD8+ ratio, representing the ratio of T helper cells to T cytotoxic cells, should also be monitored. Physiological values of the CD4+/CD8+ ratio are considered to be between 1.5 and 2.5, with some divergence across different ethnic groups, age categories, etc. (reviewed in [93]). Patients who are undergoing chemotherapy usually have a significantly higher portion of CD8+ cells [94]. CD4+ and CD8+ cells can be distinguished into several phenotype subtypes—naïve (Tn), effector (Teff), and memory (Tm) T-cells present 3 main T-cell phenotype subtypes. Memory T-cells can be further divided into long-lived central memory (Tcm) T-cell and short-lived effector memory (Tem) T-cell subtypes [95–97]. CAR T-cells derived purely from CD8+ cells demonstrated higher cytolytic activity in vitro. The Tcm subtype of CD8+ CARs showed the best survival rates in mice with induced tumors [98].

Similarly, CD4+ cells produce higher amounts of different cytokines (IFN-y, TNF-a, IL-2) and CD8+ cells, and every CD4+ phenotypic subtype overall improves the survival rates in mouse models [98]. The synergy between CD4+ and CD8+ T-cells was previously documented in mouse models [99], and the utilization of optimal CAR T-cell composition could be beneficial for patients.

## 4. Conclusion and Future Perspectives

CAR T-cell-based therapy is currently used in the treatment of hematological malignancies, and with two drugs already approved by the US FDA, the number of medicinal products with similar properties will almost certainly increase in the future. The main challenges for the broader use of CAR T-cell technology in clinical practice will probably be (i) reducing the manufacturing cost, (ii) overcoming the main safety hazards, and (iii) developing improved versions of CAR T-cells by introducing more advanced constructs. Although both viral and nonviral methods have their respective advantages and disadvantages, nonviral methods hold great potential to meet these challenges and take CAR technology beyond the field of hematological disorders.

## Figures and Tables

**Table 1:** Key differences between the available individual types of vectors used for the preparation of CAR T-cells.

Transduction method	Viral vectors	Transposon vectors	
Specifications	LV/RV vectors	Sleeping beauty	PiggyBac
Efficiency	Very high	Low-medium	Medium
Manufacture cost	High	Moderate	Moderate
Integration profile	Biased	No documented bias	Biased
Vector capacity	+/-10 kb	5 kb to tens of kb	Hundreds of kb
Stability	Stable	Stable	Stable
Manufacture support	Fully closed culture systems	Semiclosed culture systems	Semiclosed culture systems

The efficiency (and cargo capacity) of different transposon systems is variable between different mutation variants. Generally, PB systems outperform SB systems [32], with the exception of chosen hyperactive mutant variants [58]. SB vectors have the lowest inclination to integrate near proto-oncogenes, and PB vectors demonstrated observable bias [32, 43], similar to viral vectors [37]. Viral-mediated transduction is considered to be less safe due to higher affinity for integration near active transcription sites [59–61].

## Data Availability

It is a review article. The data used to support the findings are cited in the References.

## References

[1] Gross G., Waks T., Eshhar Z. (1989). Expression of immunoglobulin-T-cell receptor chimeric molecules as functional receptors with antibody-type specificity. *Proceedings of the National Academy of Sciences of the United States of America*.

[2] Porter D. L., Levine B. L., Kalos M., Bagg A., June C. H. (2011). Chimeric antigen receptor–modified T cells in chronic lymphoid leukemia. *The New England Journal of Medicine*.

[3] Kalos M., Levine B. L., Porter D. L. (2011). T cells with chimeric antigen receptors have potent antitumor effects and can establish memory in patients with advanced leukemia. *Science Translational Medicine*.

[4] Maher J., Brentjens R. J., Gunset G., Rivière I., Sadelain M. (2002). Human T-lymphocyte cytotoxicity and proliferation directed by a single chimeric TCR*ζ* /CD28 receptor. *Nature Biotechnology*.

[5] Imai C., Mihara K., Andreansky M. (2004). Chimeric receptors with 4-1BB signaling capacity provoke potent cytotoxicity against acute lymphoblastic leukemia. *Leukemia*.

[6] Till B. G., Jensen M. C., Wang J. (2012). CD20-specific adoptive immunotherapy for lymphoma using a chimeric antigen receptor with both CD28 and 4-1BB domains: pilot clinical trial results. *Blood*.

[7] Zhong X.-S., Matsushita M., Plotkin J., Riviere I., Sadelain M. (2010). Chimeric antigen receptors combining 4-1BB and CD28 signaling domains augment PI_3_kinase/AKT/Bcl-X_L_ activation and CD8^+^ T cell-mediated tumor eradication. *Molecular Therapy*.

[8] Zhao Y., Wang Q. J., Yang S. (2009). A herceptin-based chimeric antigen receptor with modified signaling domains leads to enhanced survival of transduced T lymphocytes and antitumor activity. *The Journal of Immunology*.

[9] Kerkar S. P., Muranski P., Kaiser A. (2010). Tumor-specific CD8+T cells expressing interleukin-12 eradicate established cancers in lymphodepleted hosts. *Cancer Research*.

[10] Chmielewski M., Hombach A. A., Abken H. (2014). Of CARs and TRUCKs: chimeric antigen receptor (CAR) T cells engineered with an inducible cytokine to modulate the tumor stroma. *Immunological Reviews*.

[11] Krenciute G., Prinzing B. L., Yi Z. (2017). Transgenic expression of IL15 improves antiglioma activity of IL13R*α*2-CAR T cells but results in antigen loss variants. *Cancer Immunology Research*.

[12] Avanzi M. P., Yeku O., Li X. (2018). Engineered tumor-targeted T cells mediate enhanced anti-tumor efficacy both directly and through activation of the endogenous immune system. *Cell Reports*.

[13] Hegde M., Mukherjee M., Grada Z. (2016). Tandem CAR T cells targeting HER2 and IL13R*α*2 mitigate tumor antigen escape. *The Journal of Clinical Investigation*.

[14] Morgan R. A., Boyerinas B. (2016). Genetic modification of T cells. *Biomedicine*.

[15] Fesnak A. D., June C. H., Levine B. L. (2016). Engineered T cells: the promise and challenges of cancer immunotherapy. *Nature Reviews. Cancer*.

[16] Cavazzana-Calvo M., Hacein-Bey S., de Saint Basile G. (2000). Gene therapy of human severe combined immunodeficiency (SCID)-X1 disease. *Science*.

[17] Hacein-Bey-Abina S., Garrigue A., Wang G. P. (2008). Insertional oncogenesis in 4 patients after retrovirus-mediated gene therapy of SCID-X1. *The Journal of Clinical Investigation*.

[18] Naldini L., Blomer U., Gallay P. (1996). In vivo gene delivery and stable transduction of nondividing cells by a lentiviral vector. *Science*.

[19] Jarrosson-Wuilleme L., Goujon C., Bernaud J., Rigal D., Darlix J.-L., Cimarelli A. (2006). Transduction of nondividing human macrophages with gammaretrovirus-derived vectors. *Journal of Virology*.

[20] Ciuffi A. (2008). Mechanisms lentivirus integration site selection. *Current Gene Therapy*.

[21] Ramanayake S., Bilmon I., Bishop D. (2015). Low-cost generation of Good Manufacturing Practice-grade CD19-specific chimeric antigen receptor-expressing T cells using piggyBac gene transfer and patient-derived materials. *Cytotherapy*.

[22] Bouard D., Alazard-Dany N., Cosset F.-L. (2009). Viral vectors: from virology to transgene expression. *British Journal of Pharmacology*.

[23] Singh H., Moyes J. S. E., Huls M. H., Cooper L. J. N. (2015). Manufacture of T cells using the Sleeping Beauty system to enforce expression of a CD19-specific chimeric antigen receptor. *Cancer Gene Therapy*.

[24] Chicaybam L., Abdo L., Carneiro M. (2019). CAR T cells generated using sleeping beauty transposon vectors and expanded with an EBV-transformed lymphoblastoid cell line display antitumor activity in vitro and in vivo. *Human Gene Therapy*.

[25] Yant S. R., Meuse L., Chiu W., Ivics Z., Izsvak Z., Kay M. A. (2000). Somatic integration and long-term transgene expression in normal and haemophilic mice using a DNA transposon system. *Nature Genetics*.

[26] Izsvák Z., Hackett P. B., Cooper L. J. N., Ivics Z. (2010). Translating sleeping beauty transposition into cellular therapies: victories and challenges. *BioEssays*.

[27] Geurts A. M., Yang Y., Clark K. J. (2003). Gene transfer into genomes of human cells by the sleeping beauty transposon system. *Molecular Therapy*.

[28] Jin Z., Maiti S., Huls H. (2011). The hyperactive Sleeping Beauty transposase SB100X improves the genetic modification of T cells to express a chimeric antigen receptor. *Gene Therapy*.

[29] Yant S., Wu X., Huang Y. (2004). 817. Nonrandom insertion site preferences for the SB transposon in vitro and in vivo. *Molecular Therapy*.

[30] Mitchell R. S., Beitzel B. F., Schroder A. R. W. (2004). Retroviral DNA integration: ASLV, HIV, and MLV show distinct target site preferences. *PLoS Biology*.

[31] Kebriaei P., Singh H., Huls M. H. (2016). Phase I trials using sleeping beauty to generate CD19-specific CAR T cells. *The Journal of Clinical Investigation*.

[32] Huang X., Guo H., Tammana S. (2010). Gene transfer efficiency and genome-wide integration profiling of Sleeping Beauty , Tol2 , and piggyBac transposons in human primary T cells. *Molecular Therapy*.

[33] Tsukahara T., Iwase N., Kawakami K. (2015). The Tol2 transposon system mediates the genetic engineering of T-cells with CD19-specific chimeric antigen receptors for B-cell malignancies. *Gene Therapy*.

[34] Urasaki A., Morvan G., Kawakami K. (2006). Functional dissection of the Tol 2 transposable element identified the minimal cis-sequence and a highly repetitive sequence in the subterminal region essential for transposition. *Genetics*.

[35] Cary L. C., Goebel M., Corsaro B. G., Wang H.-G., Rosen E., Fraser M. J. (1989). Transposon mutagenesis of baculoviruses: analysis of Trichoplusia ni transposon IFP2 insertions within the FP-locus of nuclear polyhedrosis viruses. *Virology*.

[36] Fraser M. J., Clszczon T., Elick T., Bauser C. (1996). Precise excision of TTAA-specific lepidopteran transposons piggyBac (IFP2) and tagalong (TFP3) from the baculovirus genome in cell lines from two species of Lepidoptera. *Insect Molecular Biology*.

[37] Galvan D. L., Nakazawa Y., Kaja A. (2009). Genome-wide mapping of PiggyBac transposon integrations in primary human T cells. *Journal of Immunotherapy*.

[38] Gogol-Döring A., Ammar I., Gupta S. (2016). Genome-wide profiling reveals remarkable parallels between insertion site selection properties of the MLV retrovirus and the piggyBac transposon in primary human CD4^+^ T cells. *Molecular Therapy*.

[39] Wu S. C.-Y., Meir Y.-J. J., Coates C. J. (2006). piggyBac is a flexible and highly active transposon as compared to sleeping beauty, Tol2, and Mos1 in mammalian cells. *Proceedings of the National Academy of Sciences of the United States of America*.

[40] Nakazawa Y., Huye L. E., Dotti G. (2009). Optimization of the PiggyBac transposon system for the sustained genetic modification of human T lymphocytes. *Journal of Immunotherapy*.

[41] Morgan R. A., Yang J. C., Kitano M., Dudley M. E., Laurencot C. M., Rosenberg S. A. (2010). Case report of a serious adverse event following the administration of T cells transduced with a chimeric antigen receptor recognizing ERBB2. *Molecular Therapy*.

[42] Yusa K., Zhou L., Li M. A., Bradley A., Craig N. L. (2011). A hyperactive piggyBac transposase for mammalian applications. *Proceedings of the National Academy of Sciences of the United States of America*.

[43] Doherty J. E., Huye L. E., Yusa K., Zhou L., Craig N. L., Wilson M. H. (2012). Hyperactive piggyBac gene transfer in human cells and in vivo. *Human Gene Therapy*.

[44] Cadiñanos J., Bradley A. (2007). Generation of an inducible and optimized piggyBac transposon system†. *Nucleic Acids Research*.

[45] Saito S., Nakazawa Y., Sueki A. (2014). Anti-leukemic potency of piggyBac -mediated CD19-specific T cells against refractory Philadelphia chromosome-positive acute lymphoblastic leukemia. *Cytotherapy*.

[46] Wang J., Lupo K. B., Chambers A. M., Matosevic S. (2018). Purinergic targeting enhances immunotherapy of CD73+ solid tumors with piggyBac-engineered chimeric antigen receptor natural killer cells. *Journal for ImmunoTherapy of Cancer*.

[47] Zhang Z., Jiang D., Yang H. (2019). Modified CAR T cells targeting membrane-proximal epitope of mesothelin enhances the antitumor function against large solid tumor. *Cell Death & Disease*.

[48] Ma Y., Chen Y., Yan L. (2020). EGFRvIII-specific CAR-T cells produced by piggyBac transposon exhibit efficient growth suppression against hepatocellular carcinoma. *International Journal of Medical Sciences*.

[49] Ptáčková P., Musil J., Štach M. (2018). A new approach to CAR T-cell gene engineering and cultivation using piggyBac transposon in the presence of IL-4, IL-7 and IL-21. *Cytotherapy*.

[50] Tseng W.-C., Haselton F. R., Giorgio T. D. (1999). Mitosis enhances transgene expression of plasmid delivered by cationic liposomes. *Biochimica et Biophysica Acta (BBA) - Gene Structure and Expression*.

[51] Karikó K., Muramatsu H., Welsh F. A. (2008). Incorporation of pseudouridine into mRNA yields superior nonimmunogenic vector with increased translational capacity and biological stability. *Molecular Therapy*.

[52] Zhao Y., Zheng Z., Cohen C. J. (2006). High - efficiency transfection of primary human and mouse T lymphocytes using RNA electroporation. *Molecular Therapy*.

[53] Harrer D. C., Simon B., Fujii S. (2017). RNA-transfection of *γ*/*δ* T cells with a chimeric antigen receptor or an *α*/*β* T-cell receptor: a safer alternative to genetically engineered *α*/*β* T cells for the immunotherapy of melanoma. *BMC Cancer*.

[54] Campillo-Davo D., Fujiki F., Van den Bergh J. M. J. (2018). Efficient and nongenotoxic RNA-based engineering of human T cells using tumor-specific T cell receptors with minimal TCR mispairing. *Frontiers in Immunology*.

[55] Mensali N., Myhre M. R., Dillard P. (2019). Preclinical assessment of transiently TCR redirected T cells for solid tumour immunotherapy. *Cancer Immunology, Immunotherapy*.

[56] Mensali N., Myhre M. R., Dillard P. (2020). Correction to: preclinical assessment of transiently TCR redirected T cells for solid tumour immunotherapy. *Cancer Immunology, Immunotherapy*.

[57] Billingsley M. M., Singh N., Ravikumar P., Zhang R., June C. H., Mitchell M. J. (2020). Ionizable lipid nanoparticle-mediated mRNA delivery for human CAR T cell engineering. *Nano Letters*.

[58] Grabundzija I., Irgang M., Mátés L. (2010). Comparative analysis of transposable element vector systems in human cells. *Molecular Therapy*.

[59] Field A.-C., Vink C., Gabriel R. (2013). Comparison of lentiviral and sleeping beauty mediated *αβ* T cell receptor gene transfer. *PLoS One*.

[60] Beard B. C., Dickerson D., Beebe K. (2007). Comparison of HIV-derived lentiviral and MLV-based gammaretroviral vector integration sites in primate repopulating cells. *Molecular Therapy*.

[61] Schröder A. R. W., Shinn P., Chen H., Berry C., Ecker J. R., Bushman F. (2002). HIV-1 integration in the human genome favors active genes and local hotspots. *Cell*.

[62] Hartmann J., Schüßler-Lenz M., Bondanza A., Buchholz C. J. (2017). Clinical development of CAR T cells—challenges and opportunities in translating innovative treatment concepts. *EMBO Molecular Medicine*.

[63] Vormittag P., Gunn R., Ghorashian S., Veraitch F. S. (2018). A guide to manufacturing CAR T cell therapies. *Current Opinion in Biotechnology*.

[64] Merten O.-W., Hebben M., Bovolenta C. (2016). Production of lentiviral vectors. *Molecular Therapy - Methods & Clinical Development*.

[65] Dull T., Zufferey R., Kelly M. (1998). A third-generation lentivirus vector with a conditional packaging system. *Journal of Virology*.

[66] Sanber K. S., Knight S. B., Stephen S. L. (2015). Construction of stable packaging cell lines for clinical lentiviral vector production. *Scientific Reports*.

[67] Turtle C. J., Hanafi L.-A., Berger C. (2016). CD19 CAR–T cells of defined CD4+: CD8+ composition in adult B cell ALL patients. *Journal of Clinical Investigation*.

[68] Kochenderfer J. N., Somerville R., Lu L. (2014). Anti-CD19 CAR T cells administered after low-dose chemotherapy can induce remissions of chemotherapy-refractory diffuse large B-cell lymphoma. *Blood*.

[69] Liu E., Marin D., Banerjee P. (2020). Use of CAR-transduced natural killer cells in CD19-positive lymphoid tumors. *The New England Journal of Medicine*.

[70] Srour S. A., Singh H., McCarty J. (2020). Long-term outcomes of Sleeping Beauty-generated CD19-specific CAR T-cell therapy for relapsed-refractory B-cell lymphomas. *Blood*.

[71] Magnani C. F., Gaipa G., Lussana F. (2020). Sleeping Beauty–engineered CAR T cells achieve antileukemic activity without severe toxicities. *The Journal of Clinical Investigation*.

[72] Singh H., Figliola M. J., Dawson M. J. (2013). Manufacture of clinical-grade CD19-specific T cells stably expressing chimeric antigen receptor using Sleeping Beauty system and artificial antigen presenting cells. *PLoS One*.

[73] Fielding A. K., Richards S. M., Chopra R. (2007). Outcome of 609 adults after relapse of acute lymphoblastic leukemia (ALL); an MRC UKALL12/ECOG 2993 study. *Blood*.

[74] Raja Manuri P. V., Wilson M. H., Maiti S. N. (2010). piggyBac transposon/transposase system to generate CD19-specific T cells for the treatment of B-lineage malignancies. *Human Gene Therapy*.

[75] Gee A. P. (2018). GMP CAR-T cell production. *Best Practice & Research Clinical Haematology*.

[76] Fernández L., Fernández A., Mirones I. (2019). GMP-compliant manufacturing of NKG2D CAR memory T cells using CliniMACS prodigy. *Frontiers in Immunology*.

[77] Mock U., Nickolay L., Philip B. (2016). Automated manufacturing of chimeric antigen receptor T cells for adoptive immunotherapy using CliniMACS prodigy. *Cytotherapy*.

[78] Rojewski M. T., Fekete N., Baila S. (2013). GMP-compliant isolation and expansion of bone marrow-derived MSCs in the closed, automated device quantum cell expansion system. *Cell Transplantation*.

[79] Coeshott C., Vang B., Jones M., Nankervis B. (2019). Large-scale expansion and characterization of CD3+ T-cells in the quantum® cell expansion system. *Journal of Translational Medicine*.

[80] Walker A., Johnson R. (2016). Commercialization of cellular immunotherapies for cancer. *Biochemical Society Transactions*.

[81] Wiesinger M., März J., Kummer M. (2019). Clinical-scale production of CAR-T cells for the treatment of melanoma patients by mRNA transfection of a CSPG4-specific CAR under full GMP compliance. *Cancers*.

[82] Bouet-Toussaint F., Genetel N., Rioux-Leclercq N. (2000). Interleukin-2 expanded lymphocytes from lymph node and tumor biopsies of human renal cell carcinoma, breast and ovarian cancer. *European Cytokine Network*.

[83] Crompton J. G., Sukumar M., Restifo N. P. (2014). Uncoupling T-cell expansion from effector differentiation in cell-based immunotherapy. *Immunological Reviews*.

[84] Kaartinen T., Luostarinen A., Maliniemi P. (2017). Low interleukin-2 concentration favors generation of early memory T cells over effector phenotypes during chimeric antigen receptor T-cell expansion. *Cytotherapy*.

[85] Sabatino M., Hu J., Sommariva M. (2016). Generation of clinical-grade CD19-specific CAR-modified CD8+ memory stem cells for the treatment of human B-cell malignancies. *Blood*.

[86] Hinrichs C. S., Spolski R., Paulos C. M. (2008). IL-2 and IL-21 confer opposing differentiation programs to CD8+ T cells for adoptive immunotherapy. *Blood*.

[87] Cieri N., Camisa B., Cocchiarella F. (2013). IL-7 and IL-15 instruct the generation of human memory stem T cells from naive precursors. *Blood*.

[88] Zhou J., Jin L., Wang F., Zhang Y., Liu B., Zhao T. (2019). Chimeric antigen receptor T (CAR-T) cells expanded with IL-7/IL-15 mediate superior antitumor effects. *Protein & Cell*.

[89] Leen A. M., Sukumaran S., Watanabe N. (2014). Reversal of tumor immune inhibition using a chimeric cytokine receptor. *Molecular Therapy*.

[90] Wang Y., Jiang H., Luo H. (2019). An IL-4/21 inverted cytokine receptor improving CAR-T cell potency in immunosuppressive solid-tumor microenvironment. *Frontiers in Immunology*.

[91] Chavez A. T., McKenna M. K., Canestrari E. (2019). Expanding CAR T cells in human platelet lysate renders T cells with in vivo longevity. *Journal for Immunotherapy of Cancer*.

[92] Stemberger C., Dreher S., Tschulik C. (2012). Novel serial positive enrichment technology enables clinical multiparameter cell sorting. *PLoS One*.

[93] McBride J. A., Striker R. (2017). Imbalance in the game of T cells: what can the CD4/CD8 T-cell ratio tell us about HIV and health?. *PLoS Pathogens*.

[94] Mackall C. L., Fleisher T. A., Brown M. R. (1995). Age, thymopoiesis, and CD4+ T-lymphocyte regeneration after intensive chemotherapy. *The New England Journal of Medicine*.

[95] Graef P., Buchholz V. R., Stemberger C. (2014). Serial transfer of single-cell-derived immunocompetence reveals stemness of CD8+ central memory T cells. *Immunity*.

[96] Buchholz V. R., Flossdorf M., Hensel I. (2013). Disparate individual fates compose robust CD8+ T cell immunity. *Science*.

[97] Gattinoni L., Lugli E., Ji Y. (2011). A human memory T cell subset with stem cell-like properties. *Nature Medicine*.

[98] Sommermeyer D., Hudecek M., Kosasih P. L. (2016). Chimeric antigen receptor-modified T cells derived from defined CD8^+^ and CD4^+^ subsets confer superior antitumor reactivity in vivo. *Leukemia*.

[99] Moeller M., Kershaw M. H., Cameron R. (2007). Sustained antigen-specific antitumor recall response mediated by gene-modified CD4+ T helper-1 and CD8+ T cells. *Cancer Research*.

